# Risk Factors and Outcomes of Surgical Site Infections of the Spine: A Retrospective Multi-Center Analysis

**DOI:** 10.3390/jcm14103520

**Published:** 2025-05-17

**Authors:** Bailey D. Lupo, Wesley P. Jameson, Christian J. Quinones, Alexandre E. Malek, Deepak Kumbhare, Bharat Guthikonda, Stanley Hoang

**Affiliations:** 1 Department of Neurosurgery, Louisiana State University Health Sciences Center, Shreveport, LA 71103, USA; 2 Division of Infectious Diseases, Department of Medicine, School of Medicine, LSU Health Shreveport, Shreveport, LA 71103, USA

**Keywords:** spinal infection, surgical site infection, spine surgery, risk factors

## Abstract

**Background/Objectives**: Surgical site infections (SSIs) in spine surgery pose significant risks, including neurological deficits, prolonged hospital stays, and increased healthcare costs. SSIs are classified by their location and include superficial, deep, and organ/space (OS) infections. In spine surgery, OS SSIs include osteomyelitis, discitis, and spinal epidural abscess. These infections are difficult to treat with conservative measures, impart significant morbidity, and incur increasing hospital costs. Despite advancements in surgical technique and infection control, the literature is conflicting on which factors are associated with a significant increase in risk of SSIs after spinal surgery. There is also a significant gap in the literature in defining the risk factors specific to OS SSIs. This study aims to identify risk factors associated with SSI after spine surgery at a single institution, as well as provide descriptive characteristics of patients with OS SSIs. **Methods**: This retrospective study analyzed spinal surgeries performed at a multi-center, single-institution between 1 January 2019 and 9 February 2025. Neurosurgical patients who underwent spine surgery were identified by ICD-10 procedure and diagnosis codes. Surgical infections were classified based on the National Healthcare Safety Network (NHSN) criteria. Univariate and multivariate analyses were performed to assess associations between patient demographics, comorbidities, and infection risk. **Results**: Of the 2363 unique spinal surgery patients, 39 developed infections, with 14 meeting the NHSN criteria for OS SSI. The overall rate of SSIs at this institution was 1.65%. Significant risk factors for developing an SSI included cardiovascular disease (*p* = 0.017) and COPD (*p* = 0.012). Multivariate analysis confirmed both risk factors identified in the univariate analysis as independent risk factors, with adjusted odds ratios of 1.97 (*p* = 0.033) and 2.072 (*p* = 0.041), respectively. The commonly cultured pathogens included *Staphylococcus aureus*, *Staphylococcus epidermidis*, and methicillin-resistant *Staphylococcus aureus*. **Conclusions**: Male sex, diabetes mellitus, gastroesophageal reflux disease, hyperlipidemia, hypertension, hardware placement, and a history of smoking were more common in patients with SSI. In the OS SSI subgroup, cardiovascular disease and COPD were associated with an increased risk of developing an OS SSI. Future research is needed to investigate more detailed risk factors and include mitigating factors of OS infection into the analysis.

## 1. Introduction

Surgical site infection (SSI) occurs in approximately 3% of patients following spine surgery [1]. Although uncommon, SSI can lead to long-lasting, devastating complications. In the most severe cases, SSI can require prolonged hospital admissions followed by long-term intravenous antibiotic therapy, necessitating frequent follow-up visits. These protracted treatments can be financially devastating, with the average cost of treating an SSI ranging from USD 15,817–USD 38,701 [2,3]. Given the rising incidence of spine surgeries, ongoing efforts to reduce SSI rates remain essential [4].

The Centers for Disease Control and Prevention (CDC) tracks the rate of SSIs in healthcare institutions through the National Healthcare Safety Network (NHSN) [5]. The NHSN provides standardized guidelines for SSI prevention as well as reporting protocols for healthcare facilities. SSI surveillance is categorized based on the depth of infection and includes superficial, deep, and organ/space (OS) infections. Superficial SSIs involve only the skin and subcutaneous tissue at the surgical site, while deep SSIs extend into the fascial and muscular layers. The third and most morbid classification of SSI is OS SSI. OS SSIs include the following pathologies: osteomyelitis, discitis, and spinal epidural abscesses (SEAs) [6]. The morbidity of OS SSI is significantly higher with SEA capable of causing neurological deficits or irreversible spinal cord injury in mere hours [7,8]. Similarly, untreated spinal osteomyelitis can predispose patients to pathological fractures, continued infection, and neurological injury [9]. The timely diagnosis of OS infections is required to optimize patient outcomes, as evidence by Chow et al., who reported 80% of patients experience neurological improvement following surgical decompression [8]. Imaging modalities are capable of detecting SEA early in the disease course [10]. However, the onset of clinical manifestations is typically insidious [10]. Therefore, the diagnosis of SEA often occurs late in the disease course [10]. By identifying risk factors, clinicians can diagnose OS SSI earlier in the disease course.

While risk factors for superficial and deep SSI have been adequately described, few studies investigate the risk factors for OS SSI. With OS SSI being significantly more morbid and rarer than their more superficial counterparts, investigation into risk factors specific to OS SSI is warranted. This study aims to analyze cases of SSI from 2019 to 2025 in a neurosurgical department to identify risk factors for all SSI and characterize OS SSIs.

## 2. Materials and Methods

### 2.1. Study Design

**Figure 1:**
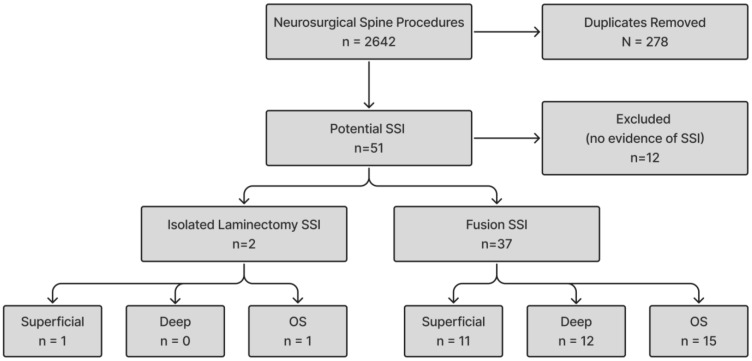
Flow chart of inclusion criteria for infections.

This retrospective study examined the risk factors and rates of surgical spine infections of spinal surgeries performed by the neurosurgery department at this institution between 1 January 2019 and 9 February 2025. The study was approved by the Louisiana State University Health Sciences Center Shreveport Institutional Review Board (LSUHSC-S IRB) (STUDY00001101) on 19 August 2019. The need for informed consent was waived due to the retrospective nature of the study. Patients undergoing any neurosurgical intervention from the multi-center, single-institution system were identified through the electronic health record (EHR) and included in this analysis. Duplicate patients were removed, and the total group was divided into laminectomies and fusions, using ICD-10 codes for the respective groups that were obtained from the National Healthcare Safety Network (NHSN) for each group. For statistical analysis, duplicate patients were deleted from the neurosurgical spine procedures group to compare unique patient risk factors. To obtain a group of laminectomies without fusions, cases that contained fusion codes were deleted from the laminectomy group. After groups of laminectomies with and without a fusion were established, infections were then identified using diagnostic codes from the NHSN that were suggestive of spinal infections. The criteria used to filter for potential SSI included ICD-10 codes suggestive of SSI (T81.40XA, T81.40XD, T81.40XS, T81.41XA, T81.41XD, T81.41XS, T81.42XA, T81.42XD, T81.42XS, T81.43XA, T81.43XD, T81.43XS, T81.44XA, T81.44XD, T81.44XS, T81.49XA, T81.49XD, and T81.49XS). Key words in the patients problem list including “Wound infection after surgery”, “Surgical site infection”, “Infection of deep incisional surgical site after procedure”, “Post-operative wound abscess”, “Superficial dehiscence of operation wound”, “Postoperative wound infection”, “Wound drainage”, “Postoperative surgical complication involving subcutaneous tissue”, and “Post-operative infection” were also used to filter for potential SSIs. This search returned 51 unique patients who were chart-reviewed for specific SSIs to determine whether it was superficial, deep, or OS, as well as to determine if a true SSI was present. We defined spinal infection of the spine in accordance with the NHSN SSI criteria. To be confirmed as a spinal infection, the patient needed to fit one of three criteria: (1) presence of purulent drainage from the spine, (2) identification of pathogenic organisms through microbiological testing of spinal tissue, or (3) evidence of an abscess or other signs of infection involving the spine as determined by imaging, anatomical examination, or histopathological analysis. The definitions of superficial, deep, and OS were also in accordance with the NHSN criteria of SSIs. For patients with SSIs of multiple levels, their deepest level of infection was included in the analysis. Spinal SSIs were defined as spinal infections occurring within 30 days of a laminectomy or within 90 days of a fusion. Chart reviewing of specific patients revealed no such evidence of an SSI and were therefore excluded from the study (*n* = 12). A visual representation of the process is included in Figure 1.

Demographics were collected for all patients using the EHR reports to identify potential risk factors for injection, including patient age, sex, body mass index (BMI), and medical history. EHR records were assessed for comorbidities, which included chronic obstructive pulmonary disease (COPD), depression, diabetes, gastroesophageal reflux disease (GERD), cardiovascular disease, hyperlipidemia (HLD), hypertension (HTN), drug abuse, and a current or past history of smoking (ever smoker). Cardiovascular disease was defined as having one or more of the following: aortic valve disorder, elevated troponin, endocarditis, acute coronary artery syndrome, congestive heart failure, coronary artery disease, and/or heart failure. HLD and hypercholesterolemia were both classified as HLD. Patients undergoing neurological surgery at the institution mentioned are given preoperative instructions for surgical site infection prevention. This surgical site infection prevention protocol includes the Association for periOperative Registered Nurses (AORN) perioperative guidelines. Recently, these institutional guidelines have been updated to include numerous pre- and perioperative infection control standards.

### 2.2. Statistical Analysis

Univariate analyses of categorical and numerical variables were performed using the Chi-square test, with Fisher’s Exact test applied when values were ≤5 in GraphPad Prism (Version 10.4.1). Categorical variables were reported as proportions, while numerical variables were reported as mean and standard deviation. Variables that were found significant in the univariate analysis were included in the subsequent multivariable logistic regression analysis conducted in R Studio (R Version 4.4.2). Significance was defined as *p*-values ≤ 0.05. Odds ratios (ORs) and adjusted odds ratios (aORs), along with their respective 95% confidence intervals (CIs), were calculated and reported.

## 3. Results

Of the 2642 total spinal neurosurgeries at this institution, 2363 of them were unique patients and were subsequently used in this analysis of risk factors for SSIs. The patients in this group had a mean age of 55.6 ± 15.7 years and a mean BMI of 30.4 ± 7.7 kg/m^2^, of which 59.2% were male. The comorbidities collected included COPD (20.0%), depression (21.1%), diabetes mellitus (DM) (47.0%), gastroesophageal reflux disease (GERD) (36.3%), cardiovascular disease (24.6%), hyperlipidemia (37.8%), hypertension (84.3%), drug abuse (0.89%), and ever smoker (39.8%) (Table 1). Surgical type was categorized based on the presence (fusion) or absence (isolated laminectomy) of implanted hardware.

In Table 1, the proportions of the risk factors of infected and non-infected are presented with the results of the univariate analysis. Out of 2363 patients who underwent spinal surgery, 39 of them had postoperative surgical site infections in accordance with the NHSN criteria. Patients with a history of COPD (19.7% vs. 35.9%, *p* = 0.012) and cardiovascular disease (24.4% vs. 41.0%, *p* < 0.001) had significantly higher rates of OS spinal infections than people without infection. Odds ratios for these risk factors were 2.275 and 2.161, respectively. No other risk factor achieved a statistically significant association with postoperative infection. In the multivariable analysis (Table 2), which included both variables that achieved statistical significance (*p*-value < 0.05), both COPD and cardiovascular disease maintained statistical significance with adjusted odds ratios of 2.072 (*p* = 0.041) and 1.97 (*p* = 0.033), respectively.

Both infection groups (laminectomy and fusion) were further subdivided by SSI category (superficial, deep, and OS). The overall rate of surgical site infections for spinal neurosurgeries at this institution was 1.65% (*n* = 39/2363). When further subdividing into the different categories of SSI, this institution had a rate of 0.47%, 0.51%, and 0.63% for superficial, deep, and OS, respectively. The OS infected group was further investigated, and descriptive characteristics, including cultured organisms, are included in Table 3. The most common cultured pathogens of OS SSIs at this institution were *Staphylococcus aureus* (*n* = 5, 2 methicillin-resistant) and *Staphylococcus epidermidis* (*n* = 2). Polymicrobial growth was identified in two patients, while no growth was identified in two patients. Surgical levels were recorded for all cases of OS SSIs involving the spine, including 2 cervical, 1 cervicothoracic, 3 thoracic, 1 thoracolumbar, 4 lumbar, and 4 lumbosacral cases.

## 4. Discussion

Surgical site infections of the spine pose significant identification and treatment challenges, leading to severe postoperative complications, including paralysis, neurological deficits, sepsis, and increased mortality. Although our study lacks statistical power, it is the first to specifically analyze OS SSIs at a single institution. In this study, we investigated the rate of SSIs of the spine at this study’s institution, the risk factors associated with SSIs of all levels, and the characteristics of OS SSIs at this institution.

### 4.1. Overall Infection Rate

While advancements in spine surgery, antiseptic techniques, and infection prevention protocols have decreased the rate of SSIs, continued investigation is needed to further lower the rate of SSIs [11]. Infection after spine surgery can result in serious complications such as treatment failure, multiple surgical debridements, increased length of antibiotic treatment, higher financial burden, and potential death [2,12]. In the literature, SSIs following spinal surgery vary widely, with rates ranging from 1.7 to 6.3% [1,13,14,15,16,17]. Notably, a study using the National Surgical Quality Improvement Program (NSQIP) reported an infection rate of overall SSI rate of 2.2% [18]. At this institution, the overall SSI rate was 1.65%, with the superficial and deep infection rates both being 0.5%. In a recent meta-analysis conducted by Zhou et al., the pooled superficial SSI rate was 1.4% [1], while other studies have reported a superficial SSI rate from 0.7% to 16.1% [19,20,21,22]. For deep SSIs, reported rates have ranged from 0.8% to 8.1%, with a pooled incidence rate of 1.5% [23]. As previously mentioned, most studies report the infection rates of all levels of SSI (superficial, deep, and OS). This study is unique in that it assesses all SSIs and divides the individual cases of OS SSI. The rates of OS SSI incidences ranged between 0.21 and 0.40% [20,24,25,26]. At this institution, 15 patients developed OS SSIs of the spine for a rate of 0.6%. These findings are slightly higher than previous studies, which could be due to multiple reasons. The first of which stems from the low number of studies reporting OS SSI, limiting accuracy. Secondly, by limiting the study to a neurosurgical specialty, the invasiveness of the procedures could vary, compared to orthopedic spine surgery, which would not involve intradural pathology. Similarly, the study was specifically designed to include only neurosurgical spine procedures; SSIs of the spine at this institution would have been excluded from this study if they were performed by a surgical specialty other than neurosurgery.

The cultured microbes identified in patients included in this study were *Staphylococcus aureus* (*n* = 3), *Staphylococcus epidermidis* (*n* = 2), methicillin-resistant *Staphylococcus aureus* (*n* = 2), *Klebsiella oxytoca* (*n* = 1), and *Enterococcus faecalis* (*n* = 1). Three of the patients with OS SSIs had no growth on culture of the surgical tissue biopsy after 96 h. Two of the patients had polymicrobial growth: one with *Serratia marcescens* and *Morganella morgani*, and the other with *Proteus mirabilis* and carbapenem-resistant organisms (CRO) *Klebsiella pneumoniae.* In the literature, most SSIs of the superficial and deep spaces include *Staphylococcus aureus*, *Staphylococcus epidermidis*, and methicillin-resistant *Staphylococcus* [1,16,19,27]. In this institutional review of the cultured microbes taken from the surgical biopsy of OS SSIs, the common pathogens seem to align with the most common reported microbes of superficial and deep SSIs. Also of note, biopsied cultures of OS SSIs of the spine were diagnostic in 12 of 15 of this study’s patients (80%). This is much higher than the reported rate of diagnostic cultures in spinal epidural abscess, which is part of the criteria for OS SSI [28]. Future studies plan to include variables that may reduce the risk of OS SSIs of the spine, including chlorohexidine gluconate (CHG) baths, minimally invasive surgery, and preoperative antibiotics, and to further investigate OS SSIs of the spine.

### 4.2. Risk Factors

Previously described risk factors associated with SSI include diabetes mellitus, obesity, coronary artery disease, advanced age, tobacco use, depression, and anemia [17,29,30,31,32,33,34,35]. Gender disparities for SSI have also been described, with female patients demonstrating a higher incidence of postoperative wound infections, while male patients experience higher rates of medical complications and postoperative mortality [18,36]. Additional risk factors for SSI and postoperative complications include depression [34] and frailty [18].

In this report, multivariate analysis for developing OS found cardiovascular disease to be significantly associated with a twofold risk of developing an SSI. The relationship between cardiovascular disease and SSI is variable and may be in part due to differing definitions of cardiovascular disease as a risk factor. De la Garza-Ramos et al. utilized a composite cardiovascular risk factor that included a history of dyspnea, percutaneous coronary intervention, and peripheral vascular disease; however, no significant association with SSI was observed [24]. Interestingly, the use of a singular cardiovascular risk factor has been associated with SSI, for example, Deng et al. examined coronary artery disease (CAD) as the sole cardiovascular risk factor and identified an eightfold increase in SSI infection frequency [37]. Likewise, Piper et al. found that patients with a history of congestive heart failure had a sixfold increase in SSI rates [20].

Several studies have reported an increased risk of SSI among patients with diabetes mellitus (DM) [13,14,29,30,38]. The increased risk has been attributed to end glycation products that impair the immune response [39]. Reported rates of diabetes among patients with SSI in previous studies have ranged from 7% to 29% [20,21,37,40]. At this institution, DM was not associated with SSI. Similarly, Piper et al. did not identify a significant association between DM and SSI, attributing this finding to variations in the definition of diabetes across studies [20]. For instance, Olsen et al. defined diabetes based on serum glucose levels exceeding 125 mg/dL preoperatively and 200 mg/dL postoperatively [38], whereas Watanabe et al. classified diabetes only in patients receiving insulin therapy and reported an odds ratio of 4.88 for SSI [13]. These discrepancies highlight the potential influence of variable diagnostic criteria on reported associations between diabetes and infection risk. Unfortunately, the level of control of DM could not be established in this study. This limitation is due to the institution included in this study being a tertiary referral hospital, where many surgical patients are referred but not directly managed within the institution. This has several limitations, which include the absence of data regarding previous diagnoses and workups made outside of the aforementioned institution.

Several prior studies have reported that elevated BMI increases postoperative infection rates. This can be attributed to the relatively avascular adipose tissue, which simultaneously applies increased pressure on local tissue, limiting blood flow and antibiotic delivery [41]. Alternatively, excess subcutaneous tissue may increase infection risk by creating dead space at the surgical site, which can serve as a nidus for bacterial colonization [42]. In our study, the BMI for all patients was 30.4 ± 7.7 and did not differ significantly between groups. Contrarily, the majority of studies have described a direct association between obesity and SSI [32,33,38,42,43]. Interestingly, Piper et al. reported increased rates of SSI in patients with a normal BMI compared to those with a BMI < 18.5 [20]. Beyond infectious complications, obesity has been linked to prolonged operative time, increased blood loss [44], extended postoperative hospital stays, and higher mortality rates [33,44].

The risk factors identified in the present study should be considered within their geographical context. The southeastern United States exhibits the highest prevalence of chronic conditions, including obesity, hypertension, hyperlipidemia, coronary heart disease, COPD, asthma, chronic kidney disease, diabetes mellitus, cancer, and depression, with reported rates of 37.8%, 13.1%, 38.8%, 36.6%, and 9.8%, respectively [45]. In comparison to the present study, hyperlipidemia remained comparable, while hypertension, COPD, and diabetes mellitus were notably higher. These marked differences in comorbidity prevalence may reflect variations in study populations, which are difficult to compare due to the degree of heterogeneity. The discrepancies with previous literature regarding BMI and DM being known risk factors for SSI may be explained by this phenomenon. Another explanation for the lack of association found between BMI and DM is the relatively high prevalence of comorbidities in the study’s geographical region, which may be a confounding factor.

### 4.3. Surgery Characteristics

The previous literature has indicated that the level and number of vertebrae manipulated during spine surgery were found to be significantly associated with rates of deep SSIs [46], with reported incidences of 3.4%, 3.7%, and 2.7% for cervical, thoracic, and lumbar spinal surgeries, respectively [1]. Investigations into OS SSIs of the spine at this institution yielded a low number of cases, limiting the statistical power. However, our study revealed that 80% (*n* = 12) of OS SSI of the spine at our institution occurred following spinal surgery at the thoracic (*n* = 3), thoracolumbar (*n* = 1), lumbar (*n* = 4) or lumbosacral (*n* = 4) levels, as opposed to 20% at the cervical/cervicothoracic level (*n* = 3). This finding is consistent with reports from the previous literature, which had suggested an increased risk of SSI for the thoracolumbar spine when compared to the cervical level [38]. A disproportionate number (*n* = 10 of 15) of the OS SSIs of the spine at this institution occurred in surgeries involving four or more levels of manipulation. Of note, all patients acquiring OS SSI of the cervical spine underwent surgery involving four or more levels of manipulation. This study suggests that operations in the thoracolumbar region and operations including four or more levels may indicate a higher risk of OS SSI of the spine. Although hardware implementation did not reach statistical significance for our study, these risk factors may also be further potentiated in patients undergoing instrumented procedures, where the incidence of SSI has been found to be significantly increased in previous literature [14].

Another critical factor influencing SSI risk is the timing of surgical intervention, as delays may contribute to increased morbidity, prolonged exposure to nosocomial pathogens, and higher mortality rates. Connor et al. reported a mean time from admission to surgery of 5.5 days, with 66.7% of cases undergoing surgery within 72 h. While this study did not include data on the time to surgical intervention, future investigations at this institution will aim to assess its impact on SSI risk, as well as additional variables such as prophylactic and intraoperative antibiotic use, chlorhexidine gluconate baths, and surgical level, along with the risk factors assessed in this study. Additionally, we plan to characterize both superficial and deep SSIs to identify potential differences in this institution’s patient population compared to the existing literature, further refining the overall understanding of infection risk factors, including surgical timing, and informing more effective prevention strategies.

The limitations of this study include its retrospective nature. Another limitation is the inevitable exclusion of some true surgical site infections due to limitations when filtering data in the electronic health record (EHR). The level of control of some of the risk factors, including DM, could not be determined; therefore, confounding variables may be present. The lack of statistical power due to the small sample size is also a limitation of this study. Additionally, due to the limited studies investigating OS SSIs, the discussion primarily compared the risk factors for overall SSI to those of OS SSIs. The low rate of SSIs and low number of OS SSIs at this institution limit the ability to draw meaningful conclusions from the study and show that future studies should include a larger patient population from multiple institutions.

## 5. Conclusions

Overall, the SSI rate at this institution was found to be 1.65%, which is well below the national average despite being in a geographical hotspot of chronic diseases in the southeast. This suggests that the risk factors associated with SSIs can be overcome. The risk factors for SSIs at this institution that were found to be associated with an increased risk of SSIs were COPD and cardiovascular disease in both univariate and multivariable logistic analyses. This evidence, along with the previous literature, suggests that both COPD and cardiovascular disease are truly associated with an increased risk of contracting a postoperative infection of the spine, a devastating complication in spinal surgery. A descriptive summary of patients with OS SSI of the spine was included in this study to better identify the risk factors associated with OS SSIs. Of these similarities, the spinal level as well as the number of vertebral levels manipulated during surgery may be associated with an increased risk of developing an OS SSI of the spine. The limitation of a small number of patients prevented us from achieving high statistical power. These findings demonstrate the need for future studies in this area of spinal infection, including investigation into risk factors and mitigating factors of infection of both all SSIs and OS SSIs individually.

## Figures and Tables

**Table 1:** Risk Factors and demographics of all patients, and univariate analysis of risk factors of all SSIs of the spine.

Comorbidity	All pts (%)	No SSI (%)	SSI (%)	*p*-Value
Patients	2363	2324	39	
Age	55.6 ± 15.7	55.7 ± 15.7	55.2 ± 14.0	0.768
BMI	30.4 ± 7.7	30.2 ± 7.6	30.4 ± 10.8	0.903
Male	1399 (59.2%)	1379 (59.3%)	20 (51.3%)	0.310
COPD	473 (20.0%)	459 (19.7%)	14 (35.9%)	**0.012**
Depression	499 (21.1%)	490 (21.0%)	9 (23.1%)	0.762
Diabetes Mellitus	1111 (47.0%)	1091 (46.9%)	20 (51.3%)	0.591
GERD	858 (36.3%)	844 (36.3%)	14 (35.9%)	0.957
CVD	582 (24.6%)	566 (24.4%)	16 (41.0%)	**0.017**
Hyperlipidemia	896 (37.8%)	880 (37.9%)	16 (41.0%)	0.687
Hypertension	1993 (84.3%)	1958 (84.2%)	35 (89.7%)	0.349
Drug Abuse	21 (0.89%)	20 (0.86%)	1 (2.5%)	0.296
Ever Smoker	941 (39.8%)	921 (39.6%)	20 (51.3%)	0.239
Hardware	2124 (89.9%)	2087 (89.8%)	37 (94.9%)	0.424

pts—patients, BMI—body mass index, COPD—chronic obstructive pulmonary disease, GERD—gastroesophageal reflux disease, CVD—cardiovascular disease, SSI—surgical site infection.

**Table 2:** Adjusted odds ratios for the risk factors included in the multivariable analysis.

Risk Factor	Adjusted OR (95% CI)	*p*-Value
COPD	2.072 (1.034–3.992)	**0.041**
Cardiovascular Disease	1.97 (1.009–3.762)	**0.033**

COPD—chronic obstructive pulmonary disease.

**Table 3:** Descriptive characteristics of OS SSIs of the spine patients.

Age	BMI	Sex	COPD	Depression	DM	GERD	CVD	HLD	HTN	Drug Abuse	Ever Smoker	Level	Surgical Tissue Culture
23	23.57	F	Yes	Yes	No	No	Yes	No	Yes	No	Yes	T1–T6	*Serratia marcescens* and *Morganella morgani*
45	41.4	M	Yes	No	No	Yes	No	No	Yes	No	Yes	L4–S1	*Proteus mirabilis*, CRO *Klebsiella pneumoniae*
64	32.23	M	Yes	No	No	Yes	No	Yes	Yes	No	no	C2–C7	Rare *Staphylococcus aureus* and *Acinetobacter baumanni* complex/hemolyticus
42	38.1	M	Yes	No	No	Yes	No	No	No	No	no	T12–L4	No growth
51	23.81	F	Yes	No	Yes	No	Yes	Yes	Yes	No	no	L3–L5	Methicillin-resistant *Staphylococcus aureus*
69	26.63	F	Yes	No	Yes	Yes	Yes	Yes	Yes	No	no	T8–T11	No growth
48	26.59	M	Yes	No	Yes	No	No	No	Yes	No	no	L4–5	*Staphylococcus epidermidis*
68	41.81	M	No	No	Yes	No	No	No	Yes	No	Yes	L2–S1	*Staphylococcus epidermidis*
62	29.53	M	No	No	No	No	No	No	No	No	No	L1–L5	*Staphylococcus aureus*
36	38.92	M	No	Yes	No	No	No	No	Yes	No	No	L4–S1	*Staphylococcus aureus*
64	27.11	F	No	No	Yes	No	No	No	Yes	No	No	C2–T2	*Klebsiella oxytoca*
61	51.54	M	Yes	No	Yes	No	Yes	No	Yes	No	No	T5–T11	*Staphylococcus aureus*
57	18.52	F	No	No	Yes	No	Yes	No	Yes	No	No	L3–L5	*Enterococcus faecalis*
67	20.98	M	No	No	No	No	No	No	No	Yes	Yes	L2–S1	No growth
72	38.52	M	No	No	Yes	No	Yes	Yes	Yes	No	Yes	C1–C5	Methicillin-resistant *Staphylococcus aureus*

BMI—body mass index, COPD—chronic obstructive pulmonary disease, CVD—cardiovascular disease, DM—diabetes mellitus, GERD—gastroesophageal reflux disease, CVD—cardiovascular disease, HLD—hyperlipidemia, HTN—hypertension, CRO—carbapenem-resistant organism.

## Data Availability

The data presented in this study are available on request from the corresponding author. The data presented in this study is restricted because it contains patient health information.

## Abbreviations

The following abbreviations are used in this manuscript:

SSI	Surgical Site Infection
OS	Organ/Space
CHG	Chlorhexidine Gluconate
IVDU	Intravenous Drug Use
SEA	Spinal Epidural Abscess
EHR	Electronic Health Record
NHSN	National Healthcare Safety Network
ICD-10	International Classification of Diseases, 10th Revision
aOR	Adjusted Odds Ratio
HLD	Hyperlipidemia
GERD	Gastroesophageal Reflux Disease
COPD	Chronic Obstructive Pulmonary Disease
NSQIP	National Surgical Quality Improvement Program
CAD	Coronary Artery Disease
OR	Odds Ratio
CoNS	Coagulase Negative Staphylococcus
DM	Diabetes Mellitus
BMI	Body Mass Index
